# Supplementary material for: Molecular Breeding of Water-Saving and Drought-Resistant Rice for Blast and Bacterial Blight Resistance
Source: Plants (Basel). 2022 Oct 8;11(19):2641. doi: 10.3390/plants11192641 (PMC9573181; doi:10.3390/plants11192641)
Supplement: Supplementary file 1 [file plants-11-02641-s001.zip › plants-1917006-supplementary.pdf]

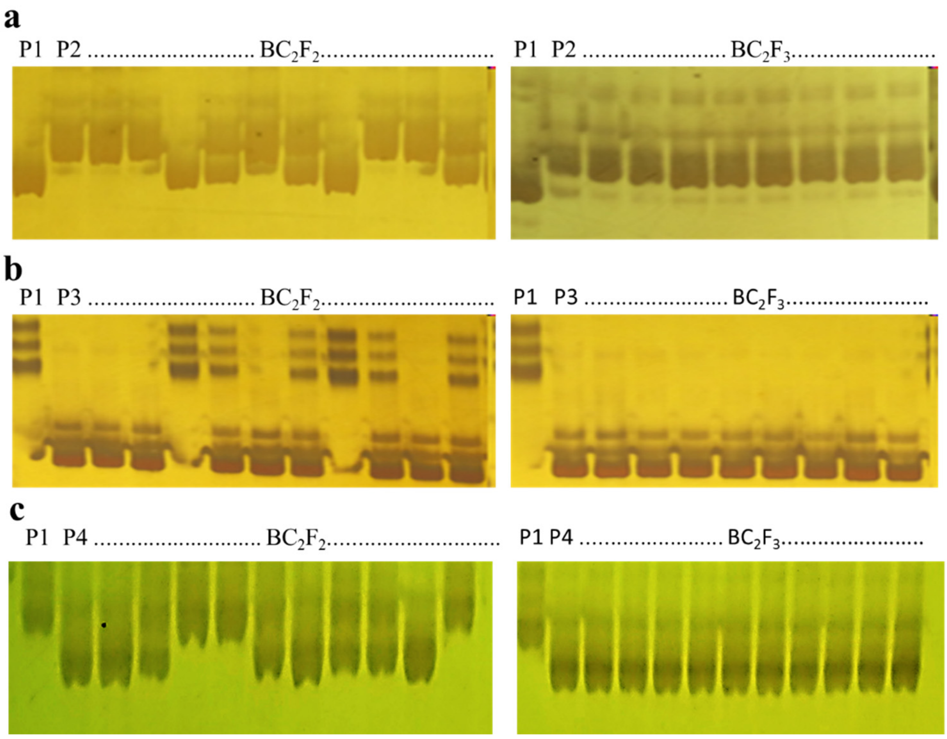


Figure S1 Foreground selection at the $BC_2F_2$ and $BC_2F_3$ generations using linked markers. Plats were screened for *Pi2* using the linked marker AP22 (**a**), and plants were screened for *Xa23* using the linked marker RM206 (**b**), and plants were screened for *xa5* using the linked marker RM611 (**c**). **a** Left panel: PCR analysis of *Pi2* in the $BC_2F_2$ generation; Right panel: PCR analysis of *Pi2* in the $BC_2F_3$ generation; **b** Left panel: PCR analysis of *Xa23* in the $BC_2F_2$ generation; Right panel: PCR analysis of *Xa23* in the $BC_2F_3$ generation. **c** Left panel: PCR analysis of *xa5* in the $BC_2F_2$ generation; Right panel: PCR analysis of *xa5* in the $BC_2F_3$ generation. P1: Hanhui3; P2: BL6; P3: CBB23; P4: IRBB5.
